# Effects of GS-967, GS-6615 and ranolazine on the responses of the rabbit aorta to adrenergic nerve stimulation

**DOI:** 10.3389/fphys.2025.1708250

**Published:** 2026-01-20

**Authors:** Maria Dolores Mauricio, Adrian Jorda, Solanye Guerra-Ojeda, Jose M. Vila, Soraya L. Valles, Martin Aldasoro

**Affiliations:** 1 Faculty of Medicina, University of Valencia, Valencia, Spain; 2 Faculty of Nursing and Podiatry, University of Valencia, Valencia, Spain

**Keywords:** adrenergic nervous stimuli, GS-6615, GS-967, late sodium current, noradrenaline, ranolazine

## Abstract

**Introduction:**

In the present study we aim to determine the effects of different inhibitors of the late sodium current (I_Na,late_) on vascular responses to adrenergic stimuli, both endogenous and exogenous.

**Methods:**

The study was performed using specific inhibitors of I_Na,late_ as GS-967, GS-6615 and Ranolazine (RAN). Rings from rabbit aorta were placed in organ baths chambers.

**Results:**

Electrical Field Stimulation (EFS) (2, 4 and 8 Hz) induced frequency-dependent contractions that were abolished by tetrodotoxin, prazosin, or guanethidine (10^−6^ M). The intervention of I_Na,late_ was observed by incubating the aortic segments with GS-967, GS-6615 or RAN. Concentration–response curves to GS-967, GS-6615 or RAN were constructed in rings precontracted with noradrenaline, endothelin-1 or KCl with or without specific inhibitors (L-NAME, nimesulide, SC-560, verapamil, nifedipine, apamin or charybdotoxin). Contraction to noradrenaline were elicited in the absence or presence of I_Na,late_ inhibitors (GS-967, GS-6615 or RAN). EFS induced frequency-dependent contractions of rings, mediated by noradrenaline acting on α_1_-adrenoceptors. I_Na,late_ blockers GS-967 and GS-6615 reduced vasoconstriction induced by sympathetic nerve stimulation, effect reversed by charybdotoxin, implicating large-conductance Ca^2+^-activated K^+^ channels. RAN elicited an attenuation of nerve-induced vasoconstriction, with 20% of this effect mediated via large-conductance Ca^2+^-activated K^+^ channels. The predominant mechanism involved competitive antagonism of RAN at α_1_-adrenergic receptors.

**Discussion:**

These findings suggest distinct mechanisms of action among I_Na,late_ blockers, highlighting the involvement of large-conductance Ca^2+^-activated K^+^ channels in GS-967 and GS-6615 effects, and a competitive α_1_-adrenoceptor antagonism for RAN. Taken together, our results indicate that GS-967, GS-6615 and RAN decrease vasoconstrictor responses due to both neural and noradrenaline-induced adrenergic stimuli. We can suggest that the use of GS-967, GS-6615 and Ranolazine may be interesting in clinical procedures involving hyperstimulation of the adrenergic nervous system.

## Graphical Abstract

**Figure:**
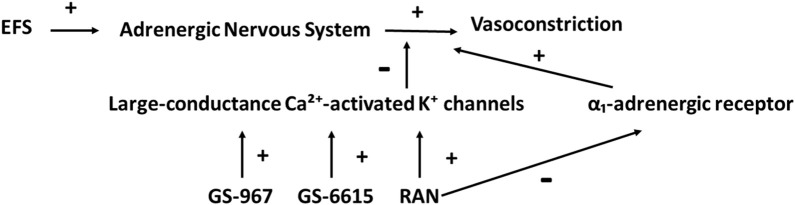

## 1 Introduction

Ranolazine (RAN), a piperazine derivative, is indicated for the treatment of refractory chronic angina (Pepine and Wolff, 1999) in combination with other anti-ischemic drugs (Chaitman, 2006; Siddiqui and Keam, 2006; Storey et al., 2020). RAN induces others cardiovascular benefices as in cardiac arrhythmias like atrial fibrillation (Chaitman et al., 2004a; Rosa et al., 2015), endothelial coronary disfunction (Deshmukh et al., 2009; Nusca et al., 2021), oxidative stress (Aldakkak et al., 2011; Dehina et al., 2014), systolic or diastolic heart failure (Chaitman et al., 2004b; Banerjee et al., 2017; Chorro et al., 2015) and hypertrophic cardiomyopathy (Cingolani et al., 2013; Mosqueira et al., 2018). Rn reduces asymmetric dimethylarginine and C-reactive protein plasma levels and increases endothelial release of vasodilator mediators like nitric oxide (Nusca et al., 2021). Moreover, it has been described metabolic effects, such as the lowering of hemoglobin A1C (HbA1c) in patients with ischemic heart disease and diabetes (Morrow et al., 2009), or the improvement of insulin secretion and β-cell survival in diabetic mice (Ning et al., 2011). RAN decreases blood glucose and glycosylated hemoglobin (HbA1c) levels (Fu et al., 2013; Lisi et al., 2019), improving cognitive aspects in patients with type 2 diabetes mellitus (Cassano et al., 2020). A recent study carried out in our laboratory demonstrates the potentiating effect of RAN on vascular sensitivity to insulin, increasing the vasodilation induced by the hormone (Guerra-Ojeda et al., 2023), reducing vascular resistance to the hormone. Also, RAN, blocking sodium channels, decreases the release of glucagon, which regulates glucose levels (Bell and Goncalves, 2021). RAN has different effects on central nervous system acting as an anticonvulsant agent (Peters et al., 20013; Park et al., 2013) and has been proposed for the treatment of neuropathic pain (Gould et al., 2009). These effects would be mediated by late INa or inwardly rectifying K^+^ current (Chen et al., 2009), enabling the development of new treatment strategies for chronic pain (Cummins et al., 2007), or epileptic disorders (Koch et al., 1998; Kahlig et al., 2010). Several studies show that RAN also has a protective role in the development of different types of dementia, mainly due to its anti-inflammatory and antioxidant effects (Aldasoro et al., 2016; Jorda et al., 2022; Samir et al., 2024).

Although the mechanism of action of RAN is not exactly known, it has been shown that at therapeutic concentrations it selectively inhibits the late inward Na^+^ current (INaL), thus reducing the intracellular concentration of Na^+^, which would inhibit the activity of the Na^+^-Ca^2+^ exchanger and the subsequent entry of Ca^2+^ into the cells (Ca^2+^i) (Kondratev et al., 2005). In this way, intracellular ionic homeostasis would be preserved, thereby reducing the tension of both ventricular and vascular muscle fibers (Antzelevitch et al., 2004; Belardinelli et al., 2006; Quinn and Kohl, 2016). RAN also acts on other cellular ion channels such as TASK-1 potassium channels, whose inhibition would contribute to its antiarrhythmic effects (Belardinelli et al., 2013; Ratte et al., 2019). In recent years, other mechanisms of action of RAN have been proposed, such as the blockade of α1-adrenergic receptors (Marchio et al., 2020) or facilitating effects on the synthesis of endothelial mediators such as NO (Deshmukh et al., 2009; Guerra-Ojeda et al., 2023). Furthermore, RAN stimulates glucose oxidation and partially reduces fatty acid oxidation (Sabbah et al., 2002), leading to a better ATP production/O_2_ consumption ratio and a decrease in H^+^, lactate and fatty acyl intermediates (McCormack et al., 1996), which provides a clear cardioprotective effect in situations of ischemia and reperfusion (Rouhana et al., 2021).

Different studies, especially those conducted in cardiac tissue, have shown that GS-967 (also called GS-458967) (Koltun et al., 2016) and GS-6615 inhibit the late sodium current (Bonatti et al., 2014; Mason and Cummins, 2020; Caves et al., 2020). There is evidence that GS-967 provides protection against ventricular arrhythmias induced by adrenergic stimuli (Alves Bento et al., 2015). It also reduces the development of catecholamine-induced atrial fibrillation (AF) (Sicouri et al., 2013), which seems to indicate that the late sodium current may be involved in the development of atrial fibrillation induced by adrenergic stimulation (Carneiro et al., 2015). On the other hand, GS-6615 (also called eleclazine), through its inhibitory effect on the late sodium current, protects against ischemia induced by adrenergic stimulation as well as atrial fibrillation induced by this process, and it reduces other consequences of adrenergic stimulation (Justo et al., 2016). By inhibiting the late sodium current, GS-6615 protects against atrial fibrillation induced by adrenergic agonists such as isoproterenol (Liu et al., 2023). However, there is no evidence that GS-967 or GS-6615 exert effects on blood vessels in humans or in other species. Investigating these two inhibitors in vascular tissue may be of pharmacological and clinical interest, given that alterations in adrenergic regulation of vascular tone can contribute to various clinical conditions.

The objective of the present study is to evaluate the effects of different inhibitors of the late sodium current on the responses of rabbit aorta to various adrenergic stimuli. Specifically, the study assesses the effects of GS-967, GS-6615, and RAN on both endogenous and exogenous adrenergic stimuli.

## 2 Materials and methods

### 2.1 Animal model

The investigation was carried out in accordance with the ethical standards in animal experimentation established by EU Directive 2010/63 and Spanish Royal Decree (RD) 1201/2005. The experiments were carried out using tissue samples according to procedure 2017/VSC/PEA/00049 type 2, authorized by the Bioethics Committee of the University of Valencia, Spain. Thirty-two male New Zealand white rabbits, weighing 3.1–3.6 kg, were used in this study and housed in a 12:12 h light/dark cycle at a constant room temperature of 22 °C and 60% humidity. Animals were euthanized following heparinization and anesthesia (sodium thiopental 60 mg/kg i.v.).

### 2.2 Preparation of vascular rings

Organ bath experiments were carried out as previously described (Aldasoro et al., 1993). Abdominal aorta was isolated and cut into 4-mm rings for isometric recording of tension. Two stainless steel L-shaped pins were introduced through the lumen of the vascular rings. One pin was fixed to the wall of the organ bath, and the other one was connected to a force-displacement transducer (FT03; Grass Instruments, West Warwick, RI, USA). Variations in isometric force were registered on a Macintosh computer (Apple Corp., Cupertino, CA, USA) using the Chart, version 7, and a MacLab/8e data acquisition system (AD Instruments). Individual rings were suspended in a 5 mL bath with a modified Krebs-Henseleit solution containing (mM) NaCl, 115; CaCl_2_, 2.5; KCl, 4.6; MgCl_2_.6H_2_O, 1.2; NaHCO_3_, 25; glucose, 11.1; and disodium EDTA, 0.01, with 95% O_2_ and 5% CO_2_ to obtain a pH 7.3–7.4, and temperature was held at 37 °C. The optimal resting tension for vascular rings was 3.5 g, and aortic preparations were allowed to equilibrate for 3 h. The contractile capacity of vascular smooth muscle was evaluated by the maximum response to KCl (60 mM). The endothelium was considered functional if relaxation to acetylcholine (10^−6^ M), in aortic rings precontracted with noradrenaline, was ≥70%. Vascular rings with dysfunctional endothelium in the control conditions were excluded.

### 2.3 Experimental procedure

#### 2.3.1 Periarterial adrenergic nerve stimulation

To obtain adrenergic nerve stimuli, electrical field stimulation (EFS) was applied through two platinum electrodes placed on both sides of the aortic segments with a 5-mm separation between the two electrodes. The electrodes were connected to a multichannel stimulator (Grass S88). The correspondence between frequency and vasomotor response was studied in a determinate range of frequencies, specifically 2, 4, and 8 Hz, with the application of 25 V stimuli (supramaximal voltage) of 0.25 ms duration for each pulse for 30 s of total duration of stimulation. The evaluation of the neurogenic nature of the contractile response to EFS was carried out by incubating the vascular segments for 15 min with tetrodotoxin (TTX) (10^−6^ M), a blocker of voltage dependent Na^+^ channels and, therefore, an inhibitor of the nerve conduction of the neural fibers present in the vascular wall; guanethidine (10^−6^ M), a blocker of the release of noradrenaline, neurotransmitter of the adrenergic nervous axons; therefore, it also inhibits adrenergic neurotransmission; or with an α_1_ adrenergic postsynaptic receptor antagonist, prazosin (10^−4^ M). Different stimulation rounds (2, 4, and 8 Hz) were provoked, with 5 min intervals between each stimulus of increasing frequency. These series of stimuli were applied again 10 min after adding TTX, guanethidine, or prazosin to the organ bath. As a control group, in another series of vascular segments, electrical field stimuli were performed without the presence of adrenergic nervous system blockers.

#### 2.3.2 Interaction between GS-967, GS-6615 and RAN with the nervous adrenergic system

The possible interaction of GS-967, GS-6615 or RAN with the sympathetic-adrenergic nervous system was assessed by incubating the vascular rings with GS-967 (10^−7^–10^−4^ M) (Mason and Cummins, 2020), GS-6615 (10^−7^–10^−4^ M) (Caves et al., 2020) or RAN (10^−7^–10^−4^ M) for 15 minutes prior to the application of adrenergic nervous stimuli at frequency of 4 Hz.

To evaluate the participation of nitric oxide (NO) in the responses to EFS, aortic rings were incubated with L-NAME (10^−4^ M) to inhibit nitric oxide synthase (eNOS). To examine the intervention of prostanoids derived from COX isoforms (COX-1 and COX-2), vascular segments were incubated with SC-560 (10^−6^ M) or nimesulide (10^−6^ M), specific blockers of COX-1 and COX-2 respectively. To establish the role of L-type voltage-dependent Ca^2+^ channels vascular samples were treated with verapamil (10^−6^ M), nifedipine (10^−6^ M), apamin (10^−6^ M) prior to EFS. The participation of Ca^2+^-activated K^+^ channels was evaluated using apamin (10^−6^ M) or charybdotoxin (10^−7^ M), antagonists of small or large conductance Ca^2+^-activated K^+^ channels respectively.

#### 2.3.3 Concentration-response curves to noradrenaline

The concentration-response curves for noradrenaline were calculated after the addiction to organ bath of cumulative doses of noradrenaline (10^−9^–10^−5^ M), so that the concentration of the agonist in the organ bath when applying successive doses is the result of the sum of the current dose with those previously administered. The following dose was administered when the maximum contractile effect was reached with the previous one. Subsequently, the different blockers, antagonists, or agonists were administered to the organ bath, normally for 15 min, before obtaining the corresponding specific concentration-response curves for each treatment. To obtain the control group, concentration-response curves were designed in other aortic segments without the presence of blockers, antagonists, or agonists.

#### 2.3.4 Interaction between RAN, GS-967, GS-6615 and exogenous noradrenaline

The analysis of the effects of RAN, GS-967, GS-6615 on the vascular responses to noradrenaline was performed by incubating the vascular segments with RAN (10^−7^–10^−4^ M), GS-967 (10^−7^–10^−4^ M) or GS-6615 (10^−7^–10^−4^ M) for 15 minutes, then proceeding to obtain the different concentration-response curves for noradrenaline. Another series of aortic segments were used as a control group in which concentration response curves to noradrenaline (10^−9^–10^−5^ M) were carried out without the presence of RAN, GS-967 or GS-6615.

#### 2.3.5 Vasodilatory effects of GS-967, GS-6615 and RAN

These effects were evaluated using different contractile agonists. Concentration-response curves to RAN (10^−7^–10^−4^ M), GS-967 (10^−7^–10^−4^ M) or GS-6615 (10^−7^–10^−4^ M) were performed in vascular rings precontracted with noradrenaline (10^−5^ M), endothelin-1 (10^−7^ M) or KCl (60 mM). In different experiments, vascular samples are incubated with L-NAME (10^−4^ M) or charybdotoxin (10^−7^ M) prior to the previous experimental procedure.

### 2.4 Drugs

The drugs used were Potassium Chloride (KCl, Merck, Darmstadt, Germany), Insulin, Acetylcholine, Noradrenaline Hydrochloride, Prazosin, Tetrodotoxin, Guanethidine, Endothelin-1, Verapamil, Nifedipine, Apamin, Charybdotoxin, Indomethacin, L-NAME (N-omega-nitro-L-arginina metil éster), Ranolazine [(+)-N-(2,6-dimetilfenil)-4 (2-hidroxi- 3-(2-metoxifenoxi)-propil)-1-piperazina acetamida], GS-967 (also called GS-458967) (triazolopyridine derivative 6-(4-(trifluoromethoxy)-phenyl)-3-(trifluoromethyl)-[1, 2, 4] triazolo [4,3-a] pyridine), GS-6615, (also called eleclazine) (3,4-dihydro-4-(2-pyrimidinylmethyl)-7-[4-(trifluoromethoxy)phenyl]-1,4-benzoxazepin-5(2H)-one) (Sigma-Aldrich, Madrid, Spain). Concentrated drug solutions were obtained with bi-distilled water, except for prazosin, which were dissolved in ethanol.

### 2.5 Statistical analysis

Descriptive analyses were performed for every study variable. Values were summarized as mean ± SEM (Standard Error of the Mean) of *n* (rabbit number) (between 9 and 12 vascular segments were used from each rabbit) aortic vessel (one for each animal). Relaxation was expressed as a percentage of the inhibition of agonist-induced response. Contraction was expressed as a percentage of the response to KCl 60 mM. EC_50_ values (concentration of agonist producing half-maximum effect) were expressed as pD_2_ (-log EC_50_) (negative logarithm of the molar concentration at which half-maximum response occurs). The normality of the data was tested with the Shapiro-Wilk test. For intergroup comparisons following a normal distribution, a one-way ANOVA was used. For multiple comparisons (post hoc), the Bonferroni test has been applied. For EFS experiments, in which the same aortic ring was used in control and experimental conditions, a paired t-test was used. The level of significance used was 5% (p < 0.05). Statistical analysis was performed using GraphPad Prism 8.3.0 (GraphPad Software, San Diego, CA, USA).

## 3 Results

### 3.1 Effects of electrical field stimulation (EFS)

**FIGURE 1:**
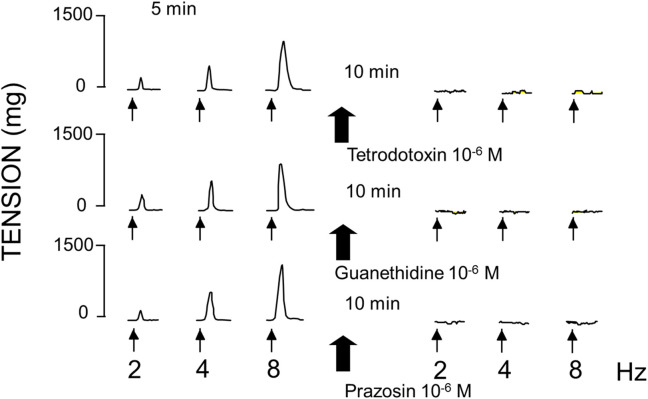
Sections of experimental records showing contractile effects of electrical field stimulation on rabbit aortic in the absence and in the presence of tetrodotoxin (10^−6^ M), guanethidine (10^−6^ M) or prazosin (10^−6^ M). Tracings are representative of various aortic rings from different rabbits. In all cases, the differences of the responses before and after treatment were statistically significant (p < 0.05).

**FIGURE 2:**
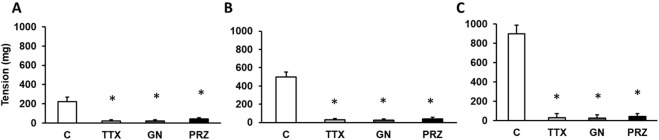
Adrenergic nervous stimulation. Effects of electrical field stimulation at 2 Hz **(A)**, 4 Hz **(B)** and 8 Hz **(C)** on rabbit aorta in the absence (control, n = 7) and in the presence of tetrodotoxin (TTX) (10^–6^ M, n = 6), guanethidine (GN) (10^–6^ M, n = 5), or prazosin (PRZ) (10^–4^ M, n = 6). Values are means ± SEM shown by vertical bars. A paired t-test was used. The level of significance used was 5% (*p < 0.05).

EFS (at 2, 4 and 8 Hz) produced frequency-dependent contractions of rabbit aortic rings at resting tension (Figure 1). Because the increases in tension induced by EFS were abolished by TTX (10^–6^ M), guanethidine (10^–6^ M), and prazosin (10^–6^ M) (Figure 2), it is assumed that the effect was due to the release of noradrenaline from adrenergic nerves acting on α_1_-adrenoceptors.

### 3.2 Effects of GS-967 or GS-6615 on the responses of the aortic segments to sympathetic nerve stimulation

**FIGURE 3:**
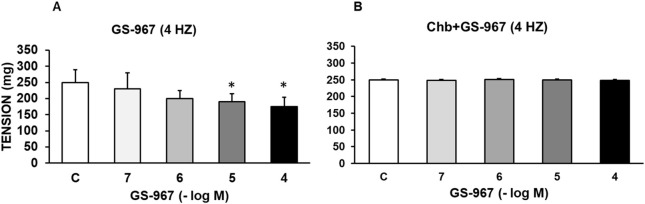
Modulatory effect of GS-967 on adrenergic nerve stimulation. Contractile effects of electrical field stimulation at 4 Hz on rabbit aorta in the absence (control, n = 8) and in the presence of GS-967 (10^−7^–10^−4^ M, n = 6) **(A)** or in vessels previously incubated with Charybdotoxin (Chb) (10^−6^ M) **(B)**. Values are means ± SEM shown by vertical bars. A paired t-test was used. The level of significance used was 5% (*p < 0.05).

**FIGURE 4 F4:**
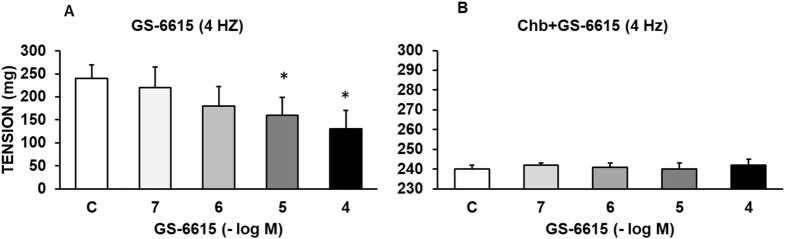
Modulatory effect of GS-6615 on adrenergic nerve stimulation. Contractile effects of electrical field stimulation at 4 Hz on rabbit aorta in the absence (control, n = 7) and in the presence of GS-6615 (10^−7^–10^−4^ M, n = 6) **(A)** or in vessels previously incubated with Charibdotoxin (Chb) (10^−6^ M) **(B)**. Values are means ± SEM shown by vertical bars. A paired t-test was used. The level of significance used was 5% (*p < 0.05).

GS-967 (10^−7^–10^−4^ M) or GS-6615 (10^−7^–10^−4^ M) caused a statistically significant reduction in the vasoconstrictor response induced by sympathetic nerve stimulation at 4 Hz (Figures 3A,4A, [Fig F4]). Charybdotoxin (10^−6^ M) prevented the inhibitory effects of GS-967 or GS-6615 (Figures 3B,4B, [Fig F4]), suggesting that large Ca^2+^-activated K^+^ channels are responsible for the antiadrenergic effects of GS-967 and GS-6615.

### 3.3 Effects of ranolazine on the responses of the aortic segments to sympathetic nerve stimulation

**FIGURE 5:**
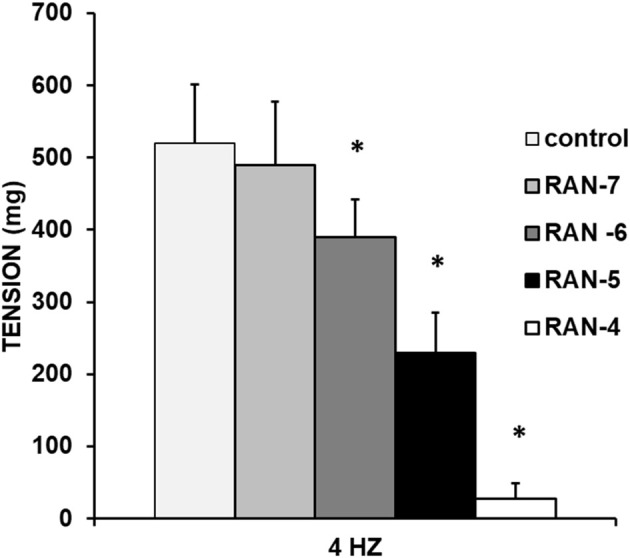
Modulatory effect of Ranolazine on adrenergic nerve stimulation. Effects of electrical field stimulation at 4 Hz on rabbit aorta in the absence (control, n = 6) and in the presence of Ranolazine (RAN) (10^−7^–10^−4^ M, n = 7). Values are means ± SEM shown by vertical bars. A paired t-test was used. The level of significance used was 5% (*p < 0.05).

Ranolazine (RAN) (10^–7^–10^–4^ M) caused a significant decrease in the vasoconstrictor response induced by sympathetic nerve stimulation at 4 Hz (Figure 5).

**FIGURE 6:**
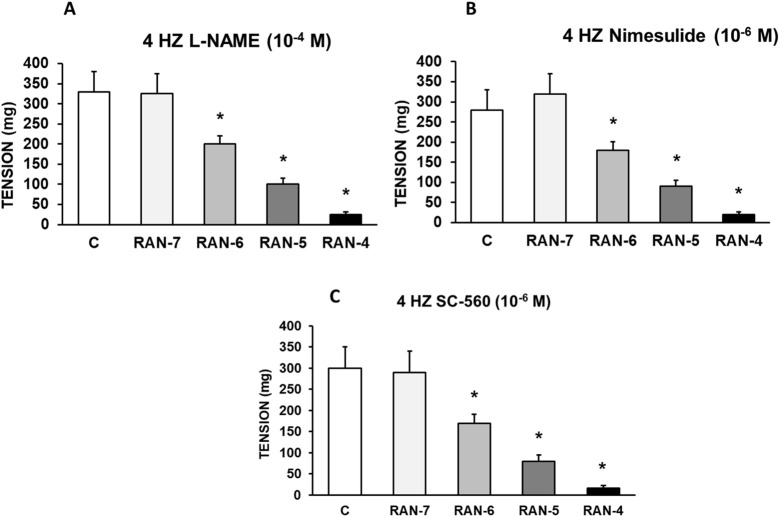
Intervention of COX-1, COX-2, and NO in the effects of RAN. Contractile effects of electrical field stimulation at 4 Hz on rabbit aorta in the absence (control, n = 7) and in the presence of Ranolazine (RAN) (10^−7^–10^−4^ M) and previously incubated with L-NAME (10^−4^ M, n = 6) **(A)** Nimesulide (10^−6^ M, n = 5) **(B)** or SC-560 (10^−6^ M, n = 5) **(C)**. Values are means ± SEM shown by vertical bars. A paired t-test was used. The level of significance used was 5% (*p < 0.05).

In Figure 6, it can be observed that the preincubation with L-NAME (10^−4^ M) (A), nimesulide (10^−6^ M) (B) or SC-560 (10^−6^ M) (C) did not cause significant changes in RAN-induced decrease in vasoconstrictor response. In this way, the participation of nitric oxide, COX-1 and COX-2 in the anti-adrenergic effects induced by RAN can be ruled out.

**FIGURE 7:**
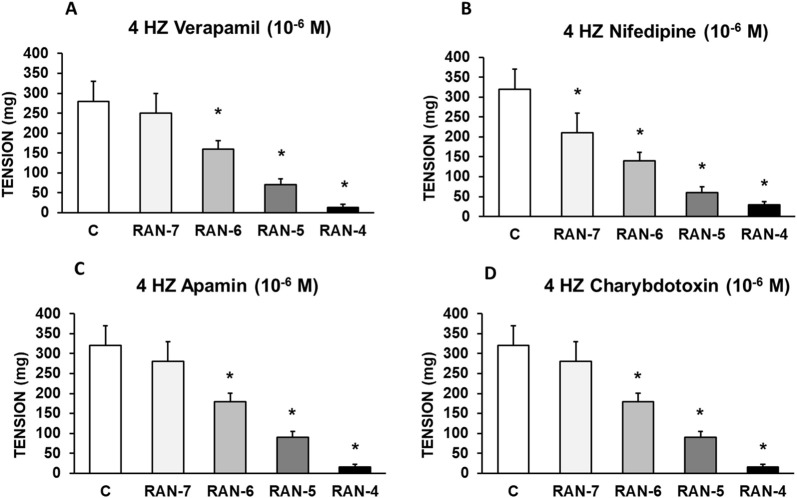
Intervention of calcium and potassium channels in the effects of RAN. Contractile effects of electrical field stimulation at 4 Hz on rabbit aorta in the absence (control, n = 7) and in the presence of Ranolazine (RAN) (10^−7^–10^−4^ M, n = 6) and previously incubated with Verapamil (10^−6^ M, n = 6) **(A)** Nifedipine (10^−6^ M, n = 6), **(B)** Apamin (10^−6^ M, n = 6) **(C)** or Charybdotoxin (10^−6^ M, n = 7) **(D)**. Values are means ± SEM shown by vertical bars. A paired t-test was used. The level of significance used was 5% (*p < 0.05).

Figure 7 shows that the pre-incubation with verapamil (10^−6^ M) (A), nifedipine (10^−6^ M) (B), apamin (10^−6^ M) (C) or charybdotoxin (10^−6^ M) (D) had no influence on RAN-induced antiadrenergic effect. The data indicates that the L-type Ca^2+^ or Ca^2+^-activated K^+^ (small or large conductance) channels are not involved in the mechanisms by which ranolazine inhibits the vascular response to adrenergic nerve stimulation.

### 3.4 Effects of RAN on the noradrenaline-induced contractions

**FIGURE 8:**
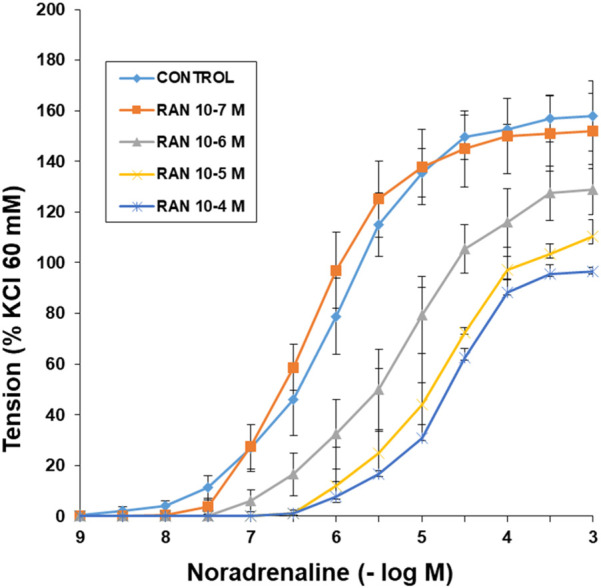
Effects of RAN on α_1_ adrenergic receptors. Concentration-response curves for noradrenaline (10^−9^–10^−3^ M) in aortic rings in the absence (control) or presence of Ranolazine (RAN) (10^−7^–10^−4^ M). Values are mean ± SEM. The data have been obtained from Table 1, where the pD2 values, Emax, and statistical significance are presented. RAN 10^−6^, 10^−5^ and 10^−4^ M with *p* < 0.05 vs. control.

Noradrenaline induces concentration-dependent vasoconstriction in rabbit aortic rings. The presence of RAN (10^–6^–10^–4^ M) shifted the noradrenaline concentration-response curves to the right in a parallel, concentration-dependent and statistically significant manner (Figure 8; Table 1).

**TABLE 1:** pD_2_ values (-log EC50) and Emax (maximum effect) to noradrenaline in rabbit aortic rings in the absence (control) and in the presence of Ranolazine (RAN) treatment. Data are shown as mean ± SEM. Emax are expressed as a percentage of response to 60 mM KCl. A one-way ANOVA was used.

Ranolazine	n	pD_2_	Emax
Control	6	6.12 ± 0.10	158 ± 11
RAN 10^-7^ M	6	6.19 ± 0.13	152 ± 12
RAN 10^-6^ M	7	5.42 ± 0,15*	129 ± 10*
RAN 10^-5^ M	7	4.8 ± 0.12*	110 ± 6*
RAN 10^-4^ M	6	4.7 ± 0.21*	96 ± 3*

*p < 0.05 vs control.

### 3.5 Effects of GS-967 and GS-6615 on the noradrenaline-induced contractions

**FIGURE 9:**
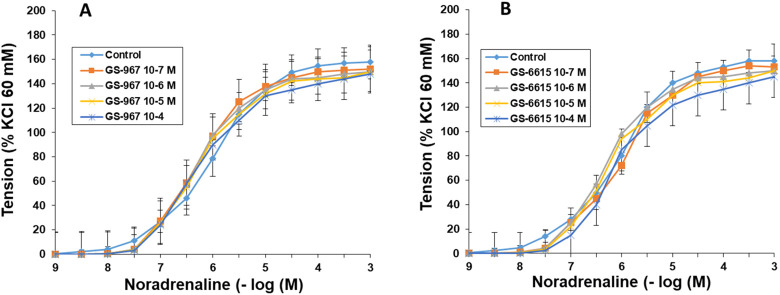
Effects of GS-967 and GS-6615 on α_1_ adrenergic receptors. Concentration-response curves for noradrenaline (10^−9^–10^−3^ M) in aortic rings in the absence (control) and in the presence of GS-967 (10^−7^–10^−4^ M) **(A)** or in the presence of GS-6615 (10^−7^–10^−4^ M) **(B)**. Values are means ± SEM. The data have been obtained from Tables 2, 3, where the pD2 values, Emax, and statistical significance are presented.

As in the previous experiments, noradrenaline induced concentration-dependent vasoconstriction in rabbit aortic segments. Neither the presence of GS-961 nor that of GS-6615 (10^–7^–10^–4^ M) shifted the noradrenaline concentration-response curves, nor did it induce changes in the maximum effect (Figures 9A,B; Tables 2, 3).

**TABLE 2:** pD_2_ values (-log EC50) and Emax (maximum effect) to noradrenaline in rabbit aortic rings in the absence (control) and in the presence of GS-967 treatment. Data are shown as mean ± SEM. Emax are expressed as a percentage of response to 60 mM KCl. A one-way ANOVA was used.

Ranolazine	n	pD_2_	Emax
Control	6	6.08 ± 0.10	157 ± 12
GS-967 10^-7^ M	7	6.25 ± 0.13	150 ± 13
GS-967 10^-6^ M	7	6.12 ± 0,15	154 ± 10
GS-967 10^-5^ M	7	6.04 ± 0.12	156 ± 9
GS-967 10^-4^ M	6	6.18 ± 0.21	148 ± 8

*p < 0.05 vs control.

**TABLE 3:** pD_2_ values (-log EC50) and Emax (maximum effect) to noradrenaline in rabbit aortic rings in the absence (control) and in the presence of GS-6615 treatment. Data are shown as mean ± SEM. Emax are expressed as a percentage of response to 60 mM KCl. A one-way ANOVA was used.

Ranolazine	n	pD_2_	Emax
Control	7	6.15 ± 0.12	158 ± 10
GS-6615 10^-7^ M	7	6.21 ± 0.11	153 ± 12
GS-6615 10^-6^ M	6	6.16 ± 0,13	154 ± 10
GS-6615 10^-5^ M	7	6.04 ± 0.13	150 ± 11
GS-6615 10^-4^ M	6	6.08 ± 0.14	145 ± 13

*p < 0.05 vs control.

### 3.6 Vasodilatory effects of RAN in the presence of different agonists

**FIGURE 10:**
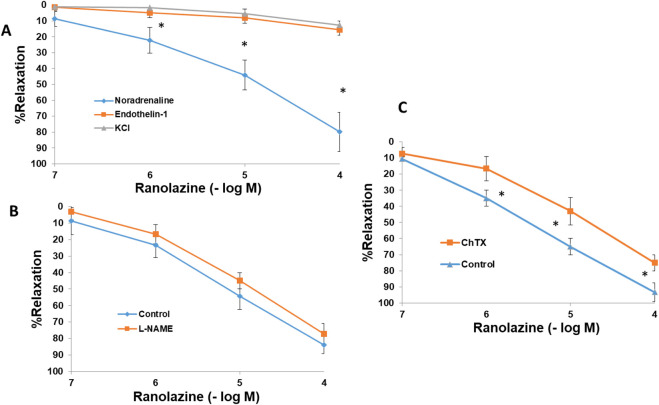
Mechanisms involved in the relaxing effects of RAN. Relaxant effects of Ranolazine (RAN) (10^−7^–10^−4^ M) on rabbit aortic rings precontracted with different agonists, noradrenaline (10^−5^ M, n = 6), endothelin-1 (10–7 M, n = 6) or KCl (60 mM, n = 5) **(A)**. Relaxant actions of Ranolazine (10^−7^–10^−4^ M) on aortic rings precontracted with noradrenaline (10^−5^ M) in the presence of L-NAME (N-omega-nitro-L-arginina metil éster) (10^−4^ M, n = 6) **(B)** or Charybdotoxin (ChTX) (10^−6^ M, n = 7) **(C)**. Values are means ± SEM. A one-way ANOVA was used (*p < 0.05).

In rings precontracted with noradrenaline (10^–5^ M), RAN (10^–7^–10^–4^ M) induced a concentration-dependent and statistically significant relaxation (80% ± 12). However, in aortic segments contracted with endothelin-1 (10^−6^ M) or KCl (60 mM) the presence of RAN did not generate any significant change in vascular tone (Figure 10A).

L-NAME (10^−4^ M) did not affect the relaxation induced by RAN which would rule out the participation of NO in this vascular effect of Rn (Figure 10B). However, in the presence of charybdotoxin (10^−6^ M) there was a decrease in the vasodilatory effect of RAN, suggesting the participation of large Ca2+-activated K+ channels in the vasodilatory response to RAN (Figure 10C).

### 3.7 Vasodilatory effects of GS-967 and GS-6615

**FIGURE 11:**
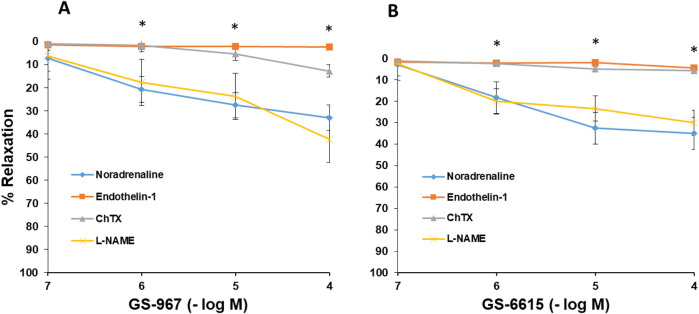
Mechanisms involved in the relaxing effects of GS-967 and GS-6615. Relaxant actions of GS-967 (10^−7^–10^−4^ M) **(A)** or GS-6615 (10^−7^–10^−4^ M) **(B)** on aortic rings precontracted with noradrenaline (10^−5^ M, n = 6) or endothelin (10^−7^ M, n = 5) in the presence of L-NAME (N-omega-nitro-L-arginina metil éster) (10^−4^ M, n = 5) or Charybdotoxin (ChTX) (10^−7^ M, n = 6). Values are means ± SEM. *p < 0.05.

In aortic rings precontracted with noradrenaline (10^–5^ M), GS-967 (10^–7^–10^–4^ M) or GS-6615 (10^–7^–10^–4^ M) induced a concentration-dependent and statistically significant relaxation. Incubation of vascular segments with L-NAME (10^−4^ M) did not cause changes in the relaxing response induced by GS-967 or GS-6615 (Figures 11A,B). As we have been able to observe in the mechanism of action of RAN, the NO does not play a role in the relaxation induced by GS-967 or GS-6615. The incubation of the aortic segments with ChTX (10^−6^ M), significantly inhibited the relaxation induced by both GS-967 and GS-6615. The inhibition of the maximum relaxing effect was 61.81% for GS-967% and 83.68% for GS-6615 (Figures 11A,B).

In another set of experiments, aortic segments were contracted with endothelin-1 (10^−6^ M). In these vascular segments, the addition of cumulative doses of GS-967 (10^−6^ -10^−4^ M) or GS-6615 (10^−6^–10^−4^ M) produced a maximum relaxing effect of 2.4% for GS-967% and 4.5% for GS-6615 (Figures 11A,B).

## 4 Discussion

Previously starting the experiments, we assessed the functional capacity of the abdominal aorta vascular segments using KCl, a non-specific vasoconstrictor (Aldasoro et al., 1993). We found that this response was like those observed in other vascular regions (Aldasoro et al., 1996). We evaluated the effects of adrenergic nervous stimulation on the rabbit aorta by using electrical field stimulation (EFS) releasing specific neurotransmitters from the adrenergic nervous synapses (Nakanishi et al., 1998). The contractile responses were abolished in the presence of tetrodotoxin, guanethidine, or prazosin. These results indicate that EFS induces vascular contraction not through a direct effect on the vascular smooth muscle, but rather by stimulating the adrenergic nerve fibers in the vascular wall. Furthermore, the fact that the contractile effect is inhibited by prazosin indicates that it is due to the release of noradrenaline from adrenergic nerve synapses, acting on postsynaptic α-1 receptors (Martinez et al., 1994). Under these conditions, we evaluated the role of the late sodium current in the responses of abdominal rabbit aorta to adrenergic stimuli, both nervous and those due to the adrenergic transmitter noradrenaline applied exogenously. We have used different antagonists of this late sodium current such as RAN, GS-967 and GS-6615.

The late sodium current blockers, RAN, GS-967 or GS-6615 inhibited the contractile responses evoked by adrenergic nerve stimuli in a concentration-dependent manner. The maximum relaxing effect achieved by RAN was 94.8%, while it was 31.2% for GS-967% and 45.8% for GS-6615. The vasodilation induced by GS-967 or GS-6615 was abolished in the presence of charybdotoxin, which seems to indicate that the late sodium current participates in the vasoconstrictor effect caused by the adrenergic nervous system and that the mechanism involved is probably the closure of calcium-activated potassium channels (Stone et al., 2010; Toischer et al., 2013). However, in our study, we were unable to determine the late Na^+^ current or the K^+^ current, so this data is subject to this limitation. In contrast, the relaxation induced by RAN was not blocked by any of the inhibitors used (L-NAME, nimesulide, SC-560, verapamil, nifedipine, apamin or charybdotoxin). The intervention of NO, COX-1, COX-2, the L-type Ca2+ or Ca2+-activated K+ (small or large conductance) channels would be ruled out in the vasodilatory response to RAN. It has been described that there are interactions between GS-967 and the adrenergic nervous system. GS-967 protects against ventricular arrhythmias induced by catecholamines (Alves Bento et al., 2015) and is also effective in reducing vulnerability to the induction of atrial fibrillation (AF) induced by catecholamines (Sicouri et al., 2013), suggesting that inhibition of the late sodium current may prevent atrial fibrillation induced by sympathetic stimulation (Carneiro et al., 2015). GS-6615, by inhibiting the late sodium current, protects against ischemia induced by adrenergic agonists as well as atrial fibrillation secondary to this process and reduces abnormalities in ventricular repolarization both in the absence and presence of associated adrenergic stimulation (Justo et al., 2016). GS-6615 inhibit isoproterenol-induced AF, demonstrating beta-adrenergic antagonism. Enhancement of late,INa represents an important factor in mediating β-adrenergic stimulation-mediated AF, which may be associated with the phosphorylation of CaMKII and NaV1.5. Inhibitions of CaMKII-late INa are effective in synergistic mode in suppressing AF associated with catecholaminergic activation (Liu et al., 2023). In the experiments where exogenous norepinephrine was used to contract the aortic segments, we observed that RAN produced a rightward shift in the concentration-response curve to norepinephrine in a concentration-dependent manner, with a reduction in the maximum effect of RAN at concentrations of 10^−5^ and 10^−4^ M. However, incubation of the aortic segments with GS-967 or GS-6615 did not produce any effect compared to the control values. This set of data suggests that RAN exerts its effect through a competitive antagonism mechanism with the adrenergic transmitter. In vascular segments contracted with norepinephrine, both RAN and GS-967 or GS-6615 induced concentration-dependent relaxation. RAN produced a maximum relaxant effect of 81%, GS-967 33%, and GS-6615 35%. However, when the vascular segments were contracted with endothelin-1 or KCl, neither RAN nor GS-967 or GS-6615 induced statistically significant vascular relaxation. This finding suggests that the relaxant effect of these three late sodium current inhibitors only occurs when the vessels are contracted with noradrenaline, likely due to a competitive mechanism with the adrenergic transmitter. On the other hand, we were able to rule out the involvement of NO in these relaxant responses, as incubation with L-NAME (a specific nitric oxide synthase inhibitor) did not produce any changes in relaxation in any of the cases. The presence of charybdotoxin reduced the relaxant response caused by RAN by 21.2%, by 85.3% for GS-967, and by 93.9% for GS-6615. From these results, we can deduce that the vascular relaxation induced by GS-967 or GS-6615 is primarily mediated by the opening of large conductance Ca2+-activated K+ channels. However, unlike the relaxation induced by GS-967 or GS-6615, the relaxant effect due to RAN was partially inhibited by charybdotoxin. Moreover, the relaxant effects induced by GS-967, GS-6615 and RAN were not altered at all by any of the antagonists (L-NAME, verapamil, nifedipine, apamin, nimesulide, SC-560) used in our research study. We used verapamil and nifedipine to assess whether calcium channels might be involved in the effects of GS-967, GS-6615, and RAN. We did not observe that incubation with verapamil or nifedipine altered the effects of GS-967, GS-6615, or RAN on adrenergic stimuli. Previously, we tested the effect of verapamil and nifedipine on the vascular response induced by EFS and observed no significant changes in this response. The mechanisms involved in vascular responses to adrenergic nerve stimulation may depend on the animal species or vessel type. In previous experiments in our laboratory, incubation with nifedipine did not alter the contractile response elicited by EFS in the human saphenous vein (Medina et al., 1998). Similarly, incubation with nifedipine did not significantly alter vascular resistance or coronary flow in lean swine at rest or during exercise; however, nifedipine significantly increased coronary blood flow and reduced coronary resistance during exercise in obese swine (Berwick et al., 2013). Therefore, the involvement of L-type calcium channel in vascular responses may depend not only on the species or vessels studied but also on other conditions such as obesity or physical exercise.

The set of results suggests that most of the effects induced by RAN are due to a different mechanism from those involved in relaxation by late sodium current blockers (Reffelmann and Kloner, 2010). In this regard, it has been proposed that this relaxant effect could be due to an antagonistic effect of RAN on the adrenergic receptors that mediate vascular contraction induced by sympathetic nerve stimulation (Létienne et al., 2001; Zhao et al., 2011). In fact, in our results, RAN did not produce relaxation in vascular segments contracted with endothelin-1 or KCl. However, when the contraction of the vascular segments was induced by noradrenaline (a neurotransmitter of the adrenergic nervous system), RAN induced a concentration-dependent relaxation, reaching a maximum effect of 81%, whereas the other late Na^+^ current inhibitors achieved a maximum relaxation of 33% (GS-967) and 35% (GS-6615) in noradrenaline-contracted segments. These data suggest that the 60% of RAN-induced relaxation that was not blocked by charybdotoxin (in contrast to what was observed with GS-967 and GS-6615) is due to an antagonistic effect of RAN reduces fatty acid oxidationon α_1_-adrenergic receptors. This mechanism of action is further supported by the fact that RAN causes a concentration-dependent rightward shift in the contractile response to noradrenaline in the vascular segments studied, an effect characteristic of drugs that act as selective antagonists at a specific receptor (Kenakin, 2014). In fact, an α_1_-adrenergic antagonistic effect of RAN has been previously described (Marchio et al., 2020). However, incubation with GS-967 or GS-6615 did not induce any shift in the contractile curve generated by noradrenaline, suggesting that the relaxant effects of these two drugs do not appear to be due to an antagonistic effect on α_1_-adrenergic receptors (Virsolvy et al., 2015) instead, to a mechanism primarily involving in the large-conductance Ca^2+^-activated K^+^ channels.

As we have indicated, GS-967 and GS-6615 reduce vasoconstriction induced by adrenergic nerve stimulation. Just as the vasoconstriction induced by norepinephrine, although they do not cause a shift in the concentration-response curve generated by this neurotransmitter. It is likely that this inhibition of the vasoconstrictor effect elicited by adrenergic nerve stimulation is related to interactions with other neurotransmitters released from adrenergic nerve endings, such as dopamine, NPY, angiotensin II, or ATP (Wier et al., 2009; Macarthur et al., 2011). We did not specifically assess these potential interactions with each of these neurotransmitters in our study, although evaluating them in future studies would be of interest.

The set of our results could have a clear clinical projection, as we have demonstrated that the three late current inhibitors used in the study have clear antiadrenergic effects. This could be relevant for pathologies where there is adrenergic overstimulation, such as different types of arterial hypertension and ischemic processes, whether myocardial or in other locations, and even in the treatment of type 2 diabetes. As we demonstrated in previous studies (Guerra-Ojeda et al., 2023), ranolazine clearly increases vascular sensitivity to insulin by reducing tissue resistance to the hormone, a crucial aspect in the pathophysiology of type 2 diabetes. It would be interesting to explore the effects of GS-967 and GS-9915 in this regard.

As a limitation of our study, it should be noted that our conclusions are based solely on pharmacological data. We did not employ electrophysiological techniques to directly measure ionic currents, which would have provided more precise insight into the involvement of both the late sodium current and the calcium-dependent potassium current in the vascular effects of adrenergic stimulation. Another possible limitation of our study is the translation of the results obtained in rabbit vessels to human vessels. In this regard, in our laboratory, we have analyzed the effects of RAN on the human saphenous vein, and the results were similar, although it was a very preliminary study (Marchio et al., 2020).

## 5 Conclusion

In conclusion, our results indicate that the sodium late current blockers GS-967 or GS-6615 reduce the adrenergic vasoconstrictor tone. This effect is likely due to the opening of large-conductance Ca^2+^-activated K^+^ channels, as its inhibition by charybdotoxin almost completely abolishes it. Moreover, no antagonistic interaction with vascular adrenergic receptors appears to be involved. However, ranolazine inhibits the vasoconstrictor effects induced by adrenergic stimuli. This effect may be due, in part, to the blockade of the late sodium current, as well as to the antagonism exerted by RAN on α_1_-adrenergic receptors. This suggests that different late Na^+^ current blockers may exert their actions through distinct mechanisms.

## Data Availability

The raw data supporting the conclusions of this article will be made available by the authors, without undue reservation.
